# Preparation and characterization of hybrid polypyrrole nanoparticles as a conducting polymer with controllable size

**DOI:** 10.1038/s41598-024-61587-1

**Published:** 2024-05-22

**Authors:** Javeed Mahmood, Nasser Arsalani, Samin Naghash-Hamed, Zahid Hanif, Kurt E. Geckeler

**Affiliations:** 1 Advanced Membranes and Porous Materials Center (AMPMC), Physical Sciences and Engineering (PSE), King Abdullah University of Science and Technology (KAUST), 23955 Thuwal, Saudi Arabia; 2 Research Laboratory of Polymer, Department of Organic and Biochemistry, Faculty of Chemistry, University of Tabriz, Tabriz, Iran; 3 School of Mechanical Engineering, Korea University of Technology and Education (KOREATECH), Cheonan, Chungnam 31253 South Korea; 4 Advanced Technology Research Centre, Korea University of Technology and Education, P.O. Box 31253, Cheonan, Chungnam Republic of Korea; 5 Department of Nanobio Materials and Electronics, Gwangju Institute of Science and Technology (GIST), Gwangju, 500712 South Korea; 6 School of Materials Science and Engineering, Gwangju Institute of Science and Technology (GIST), Gwangju, 500712 South Korea

**Keywords:** Conducting polymers, Polypyrrole, Controllable size NPs, PolyEthylenimine, Hybrid Nanoparticles, Chemistry, Materials science, Optics and photonics

## Abstract

Hybrid polypyrrole (PPy) nanoparticles were prepared using a low-temperature oxidative polymerization process in an acidic solution with polyethyleneimine (PEI) as a template and amine source. The results showed that the nanoparticles have an amorphous structure in the X-ray diffractogram and exhibited good dispersibility in water, uniform size, and a specific conductivity ranging from 0.1 to 6.9 S/cm. The particle size could be tuned from 85 to 300 nm by varying the reactant concentration. Undoping the samples with sodium hydroxide (NaOH) solution altered the optical absorption properties and surface roughness of the particles. However, it did not affect the particle size. The nanoparticles also exhibited optical sensing properties based on their UV–vis absorption changes with the pH. Moreover, nanoparticles could have potential applications in gene delivery and bio-adsorption for contaminant removal. This work demonstrates a simple and effective method for preparing hybrid polypyrrole nanoparticles with controllable size, dispersibility, and conductivity for various nanotechnology, biotechnology, and environmental engineering purposes.

## Introduction

Polymers were considered electrical insulators before the invention of conducting polymers. Conductive or conjugated polymers similar to those of inorganic semiconductors and metals have unique electrical and optical properties^1,2^. These properties are due to the alternating single and double bonds in the conjugated carbon chain, which create highly nonlocal, polarizable, electron-dense π bonds responsible for their electrical and optical behavior^3^. Biocompatibility, electrochemical activity, mechanical elasticity, electrical conductivity, and environmental stability of conductive polymers are among the desirable properties. Among a variety of conducting polymers, polythiophene (PTH), polypyrrole (PPy), polyfuran (PF), poly (3,4-ethylenedioxythiophene) (PEDOT), and poly (para-phenylene) (PPP), polyaniline (PANI), and poly (phenylenevinylene) (PPV) are mainly applied due to their high technological potential, which has been exploited in the design of rechargeable batteries, sensors, corrosion preventing coatings, biosensors, solar cells, supercapacitors, and electromagnetic shielding^4^. A two-phase system can be an effective strategy for synthesizing polymers with desired properties. By separating the reaction into two phases, it may be possible to control the reaction conditions and improve the efficiency of the synthesis process. Additionally, the formation of micelles can provide a stable environment for the oxidation of monomers and subsequent polymer growth, allowing for better control over particle size and morphology^5^.

PPy is particularly interesting due to its high stability and conductivity, ease of forming homopolymers and composites, and unique properties such as fast charge/discharge process, easy synthesis, high conductivity, flexibility, high energy density, high mass density, and low cost^6^. PPy is synthesized through various methods, such as chemical oxidative polymerization, ultrasonic radiation, electrochemical polymerization, vapor phase polymerization, electrospinning, microemulsion polymerization, mechanochemical polymerization, and photopolymerization^7^. PPy, a black powdery substance, was initially created through the chemical oxidation of a pyrrole monomer using hydrogen peroxide. In its undoped pristine state, polypyrrole exhibits insulating properties. However, when doped with halogenic electron acceptors like bromine or iodine, it demonstrates a consistent conductivity of 10^–5^ S/m^3,8^. Although it is non-crystalline and behaves in an amorphous manner, bulk polypyrrole contains 15% crystallinity, with the crystalline portion being in the monoclinic phase. The color of electrochemically synthesized PPy is yellow-black and has a thickness of 1 μm. As the protonation concentration increases, the polymer undergoes transformation and becomes more stable in air and thermally stable up to 300 °C. However, thermal degradation can occur due to the loss of impurity anions^3^. PPy exists as a conducting polymer with a positive charge in its oxidized form. However, excessive oxidation results in a loss of conductivity and charge^9^. Scanning Electron Microscope (SEM) takes pictures of PPy with different morphologies such as thin layer, nanoparticle, thick layer, nanotube, sponge-like structure, and nanofiber^10,11^.

Chemical oxidative polymerization is a simple and fast method for obtaining large amounts of PPy fine powders^12^. In general, pyrrole (PY) polymerization occurs through the oxidation of PY monomer by chemical oxidants in aqueous and non-aqueous environments^13^. Recently, several reports have been published on the polymerization of PPy with different methods. For example, Wysocka-Zołopa et al.^14^ produced PPy thin films with vertically aligned cone-like structures through the electrochemical polymerization of PY in aqueous solutions containing NaClO_4_ and polymeric or anionic surfactants such as PVP and SDS. Carbon dot-initiated PPy and CuO composites were synthesized into PPy@CuO using a simple one-step sonochemical approach by Maruthapandi et al.^15^, and their antibacterial activity was examined. A standard method in the chemical oxidative polymerization of conductive PPy involves the preparation of two separate solutions (one containing the monomer and the other, the oxidant), which are subsequently mixed to initiate the polymerization. However, it is not easy to achieve uniform structure and film thickness control for PPy prepared by this method^7^. Many studies have been conducted on the synthesis of PPy-PEI and its application in various fields. For instance, Birniwa et al.^16^ successfully synthesized a PPy-PEI polymer to remove methylene blue (MB) from aqueous solution. The results of their research showed that the prepared PPy-PEI nano-adsorbent could completely remove cationic dyes from solution. In another study, Prissanaroon-Ouajai et al.^17^ conducted a study on preparing a hybrid film of PPy/PEI and using it as a potentiometric transducer in urea biosensors. The results of measuring urea, blood, and urine in standard solutions using the PPy/PEI hybrid film showed a good correlation with the results obtained using a spectrophotometric method.

The electrical conductivity of PPy is affected by several critical factors, such as pyrrole amounts, doping level, molar ratio of O (oxidant)/pyrrole (monomer), oxidants, polymerization temperature, presence of impurities, and polymerization time^13^. In recent years, several reports have focused on the effect of various factors on the polymerization of PPy. For instance, Yusuf et al.^18^ conducted a study on the impact of FeCl_3_ and APS as oxidants on the electrical properties of PPy particles. They found that FeCl_3_ has better electrical performance than APS at room temperature. PPy particles prepared using FeCl_3_ revealed a lower resistance of about 60 Ω compared to (about 70 Ω) for APS. Effati et al.^19^ investigated the effect of the APS/PY molar ratio on the of PPy nanoparticles (NPs). They obtained resistance values of 218.78 and 152.17 Ω as APS/PY increased from 1 to 2, indicating higher conductivity of PPy NPs at an APS/PY molar ratio of 2. Majumdar et al.^20^ analyzed the impact of different impurities (HCl, CSA, and PTSA) on PPy film coated on FP substrate conductivity. The outcomes represented that the conductivity of PPy film increases from 1.78 × 10^–5^ S/cm to 3.34 × 10^–5^, 2.49 × 10^–5^, and 2.30 × 10^–5^ S/cm using HCl, CSA, and PTSA dopants, sequentially. Minisi et al.^21^ assessed the conductivity of PPy nanotubes under the influence of reaction temperature. The conductivity of PPy nanotubes increases from 64–84 to 104 S/cm as the temperature decreases from 20 to − 50 °C.

Based on the literature review conducted, it has been observed that most studies in this field use complex and time-consuming methods to synthesize and prepare PPy. The PPy nanoparticles synthesized and reported in other works are often not cost-effective and require scarce raw materials. Furthermore, no study has yet explored the impact of varying PPy amounts, [PEI]/[PY] and [FeCl3]/[PY] on particle size, yield, and conductivity. The properties of the resulting PPy-PEI NPs including conductivity, high dispersibility, surface area, and amine content, indicate potential applications in fields such as ink and paint production, removal of contaminants, and biosensors. One important aspect is that SEM and TEM analyses were conducted to measure the different nanoparticle sizes, which were obtained due to their controllable size.

The study presents a modified route for synthesizing polypyrrole nanoparticles with controllable size depending on the reaction conditions. The process involved an unstirred solution and a one-step addition of the oxidant. The resulting product was a conducting polymer of polypyrrole and hyper-branched polymer polyethyleneimine (PEI), forming PPy-PEI. Overall, this work is needed to address the limitations, improve the understanding, and advance the applications of PPy/PEI nanocomposites. It fills gaps in the existing literature, provides valuable insights, and offers opportunities for further research and development in this area.

## Experiments

### Materials

Pyrrole (96%) and methanol (99.8%) were purchased from *Fluka*. Iron (III) chloride (99%) and HCl (37%) were procured from *Merck*. Poly (ethyleneimine) was prepared from *Sigma-Aldrich*. Pyrrole was distilled under vacuum before being used to prepare PPy.

#### Apparatus

The specimens were analyzed by FT-IR analysis to determine their functional groups^22–24^. In the range of 400–4000 cm^−1^, infrared (IR) spectra were recorded on a Perkin-Elmer System 2000 (KBr pellets) (Perkin Elmer, Waltham, MA). X-ray diffraction (XRD) analysis was used to study the crystallites of doped and undoped PPy. A Rigaku x-ray diffractometer was used to measure x-ray diffraction (XRD), with the XRD patterns scanned over the angular radius of 2θ = 10–50°. The morphology of the PPY hybrid nanoparticles was analyzed using field emission scanning electron microscopy (FE-SEM, HITACHS-4700) and transmission electron microscopy (TEM, JEOL, JEM-2100, Japan). The electrical conductivity of samples was measured at room temperature using the standard four-point probe technique on compressed pellets. Conductivity measurements provide information about the electrical properties of materials. In the case of our hybrid PPy-PEI material, conductivity measurements can help assess the ability of the material to conduct electricity, which is crucial for applications such as sensors, electronic devices, and energy storage systems. The exact pH of the solution was determined using a pH meter (Mettler Toledo, Shanghai). pH measurements are essential for understanding the chemical properties and stability of materials in different environments. By measuring the pH of the hybrid PPy-PEI dispersions, we can evaluate the acidity or basicity of the material and its potential interactions with other substances or biological systems. Ultraviolet–visible (UV–Vis) was conducted on a Perkin-Elmer (Lambda 750, USA). UV–Vis spectroscopy is a technique used to study the absorption and transmission of light by materials. By analyzing the UV–Vis spectra of the Hybrid PPy-PEI samples, we can gain insights into the electronic transitions within the polymer matrix, which is essential for understanding the optical properties and electronic structure of the material. A heating rate of 10 °C/min was used for thermogravimetric analysis (TGA), which was performed with a Perkin-Elmer STA6000 (Perkin-Elmer SCIEX, Waltham, MA, USA) thermal analyzer (System 4). TGA is a thermal analysis technique used to investigate the thermal stability and decomposition behavior of materials, which are important for assessing the suitability of the material for high-temperature applications.

#### Preparation of PPy-PEI

**Figure 1:**
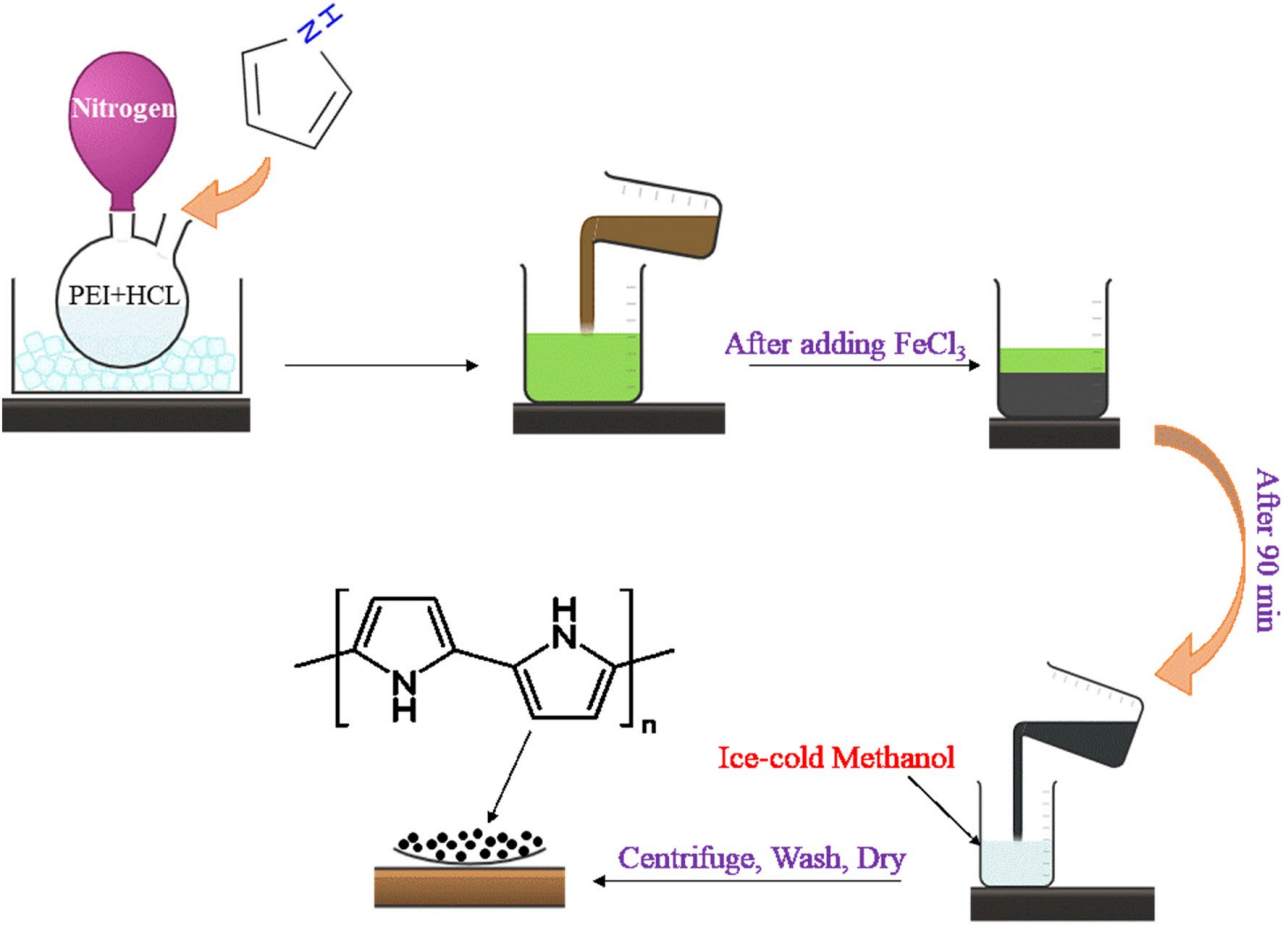
The step-by-step preparation process of PPy-PEI.

Hybrid PPy-PEI were prepared through an oxidative polymerization process at low temperatures in an acidic solution, as shown in Fig. 1. The process utilized PEI as a template and amine source and was conducted under unstirred conditions. The preparation involved dissolving 0.1 g (1.75 mmol) of PEI in 10 mL of 1 M HCl, which was placed in a round-bottomed flask in an ice bath under nitrogen gas condition. Then, 0.5 ml (7.02 mmol) of freshly distilled pyrrole was added, followed by adding 0.56 g (3.5 mmol) of FeCl_3_ solution without stirring. The mixture rapidly changed color from green to black, and after 90 min, it was poured into 0.5 L of ice-cold methanol. The black precipitate was separated by centrifugation three times. Afterward, nanoparticles were washed with methanol and dried in a vacuum. Different molar ratios were examined to prepare the nanoparticles, as listed in Table 1.

**Table 1:** Experimental conditions and results for preparing PPy NPs (PEI 0.25 g, 4.375 mmol, total volume 50 mL 1 M HCl, and reaction time 90 min in an ice bath).

Sample	Pyrrole /mL (mmol)	[PEI]/[PY]	[FeCl_3_]/[PY]	Yield (g)	Particle size (nm)	Conductivity (S/cm)
PPy-PEI1^a^	1.20 (17.27)	0.25	0.5	0.38	250–300 (SEM)	0.0364
PPy-PEI1	1.20 (17.27)	0.25	0.5	0.40	250–300 (SEM)	1.176
PPy-PEI2	0.6 (8.635)	0.5	1	0.37	–	2.50
PPy-PEI3	0.3 (4.317)	1	2	0.41	125–150 (SEM) 110–125 (TEM)	2.53
PPy-PEI3U^b^	0.3 (4.317)	1	2	25% mass reduction	125–150 (SEM) 110–125 (TEM)	0.0006
PPy (Ref)	0.3 (4.317)	0	2	0.19	350–500 (SEM)	1.10
PPy-PEI4^c^	0.3 (4.317)	1	2	0.42	120–130 (SEM) 100–110 (TEM)	6.36
PPy-PEI5	0.15 (2.158)	2	2	0.31	110–120 (SEM) 85–95 (TEM)	6.90

^a^Reaction with stirring, ^b^Undoped by 1 M NaOH, ^c^100 ml total volume.

##### Formation of the PPy-PEI

**Figure 2:**
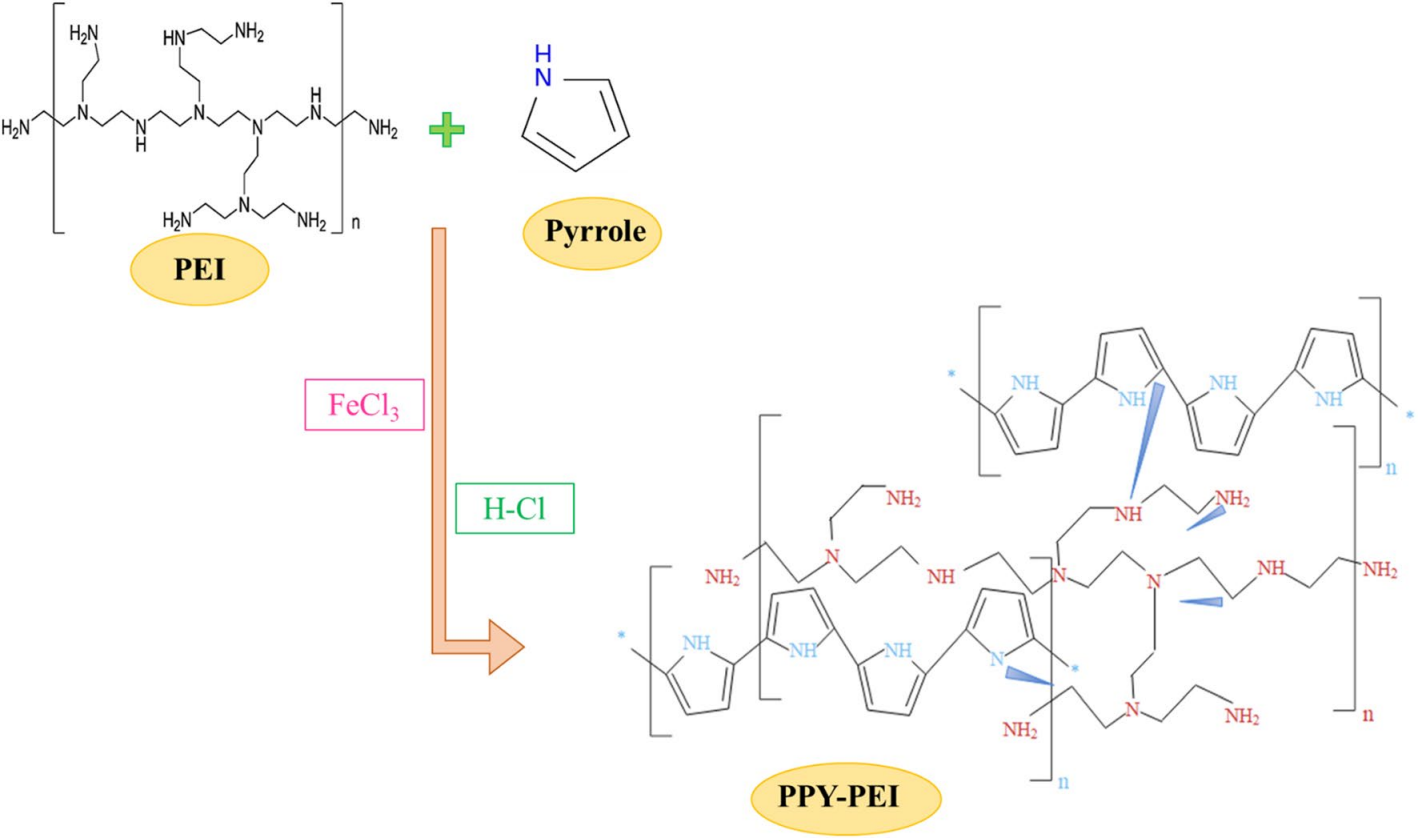
The formation of the as-prepared PPy-PEI.

The formation of PPy-PEI is illustrated in Fig. 2. The reaction begins with the initiation step, where the oxidant, typically an oxidizing agent such as FeCl_3_, reacts with pyrrole monomers. This leads to the formation of a radical species that can further react with other pyrrole monomers. The radical species generated in the initiation step undergoes polymerization, reacting with additional pyrrole monomers. This results in the growth of the polymer chain, with the pyrrole units linked together through covalent bonds. PEI molecules can be incorporated into the PPy chain during polymerization. This incorporation occurs through electrostatic interactions between the positively charged nitrogen atoms in PEI and the negatively charged sites on the PPy chain. This doping process enhances the electrical conductivity of the resulting PPy-PEI polymer^25^.

#### Preparation of the reference polypyrrole

Preparation of reference PPy was performed as described in the previous section. The addition of amine polymer was not carried out for this section. Doped PPY-PEI is poured into a 50 ml basic solution (1 M NaOH) and stirred for two hours. Then, it is filtered or centrifuged to separate the solid undoped PPy-PEI. The solid is washed with distilled water and methanol and finally dried at 50–60 °C. The experimental conditions and results for preparing PPy NPs are as follows: total volume 50 mL 1 M HCl, and reaction time 90 min in an ice bath. This incorporation occurs through electrostatic interactions between the positively charged nitrogen atoms in PEI and the nitrogen on the PPy chain. This doping process enhances the electrical conductivity of the resulting PPy-PEI polymer.

#### Preparation of samples for SEM and TEM analyses

The particle size, agglomeration, and shape of the nanoparticles were measured by FE-SEM and TEM. For FE-SEM measurements, the washed and vacuum-oven-dried PPy powders were scattered on adhesive carbon tapes, which were then mounted on copper microstubs. The powders were platinum sputter-coated for 2 min using a sputter coater. The TEM samples were prepared by drying drops of a dilute dispersion on a carbon film.

## Results and discussion

### Fourier-transform infrared spectroscopy (FTIR) analysis

**Figure 3:**
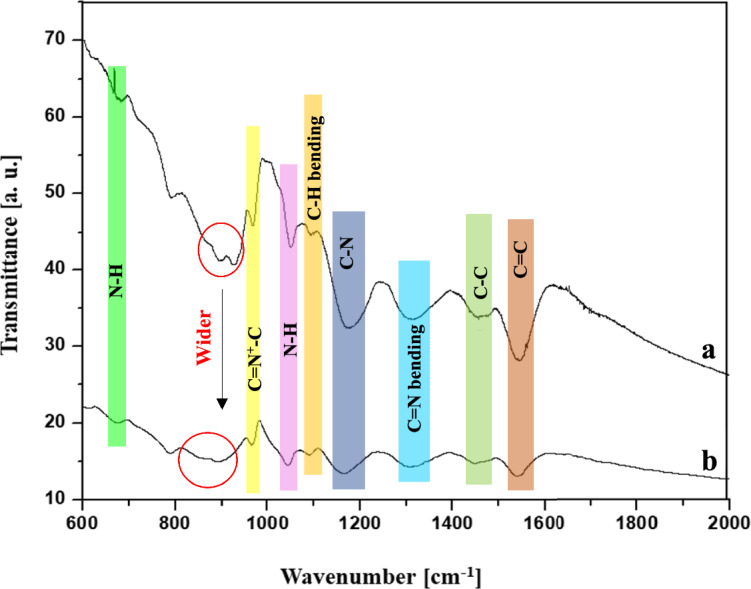
FT-IR spectra of the (**a**) reference PPy, (**b**) PPy-PEI3 NPs prepared at the same oxidant concentration, temperature, and reaction time.

Figure 3. shows the FTIR spectra of the reference PPy (Fig. 3a) and hybrid PPy NPs (PPy-PEI3) (Fig. 3b) that were prepared after 1.5 h reaction time. The samples for this study were prepared by mixing the measured quantities of the powders with KBr and then preparing the pellets. No significant peaks were located between 400–600 cm^−1^ and 2000–4000 cm^−1^, so the spectral range was set at 600–2000 cm^−1^. Clear characteristic peaks of PPy NPs can be observed from 600 to 2000 cm^−1^. The absorption bands at 1553 cm^−1^ and 1489 cm^−1^ corresponded to the C=C and C–C stretching vibration of the PY rings, respectively^26,27^. The bands seen at 1334 cm^−1^ and 1180 cm^−1^ can be attributed sequentially to the C=N bending and C–N stretching vibration. The band at about 1052 cm^−1^ is ascribed to the C–H bending vibration deformation. The C=N^+^–C stretching vibration was evidentially seen at 970 cm^−1^^28–30^. The bands observed at 1010 cm^−1^ and 676 cm^−1^ might belong to the out-of-plane ring deformation and N–H vibrations in the polymer. Aromatic ring bending vibrations were seen at 790 cm^−1^. In the 4000–2000 cm^−1^ region, which is not presented here, an absorption band at 3430 cm^−1^ was observed for all PPy hybrid nanoparticles, which can be assigned to the N–H stretching vibration mode of the PEI and PPy chains^31,32^. For the reference PPy sample, the N–H stretching band did not appear because it was in the doped state, and the tail of the electronic transition band masked the vibration. The weak band at 2890 cm^−1^ is attributed to PEI’s C–H stretching^33–36^.

### X-ray diffraction (XRD) analysis

**Figure 4:**
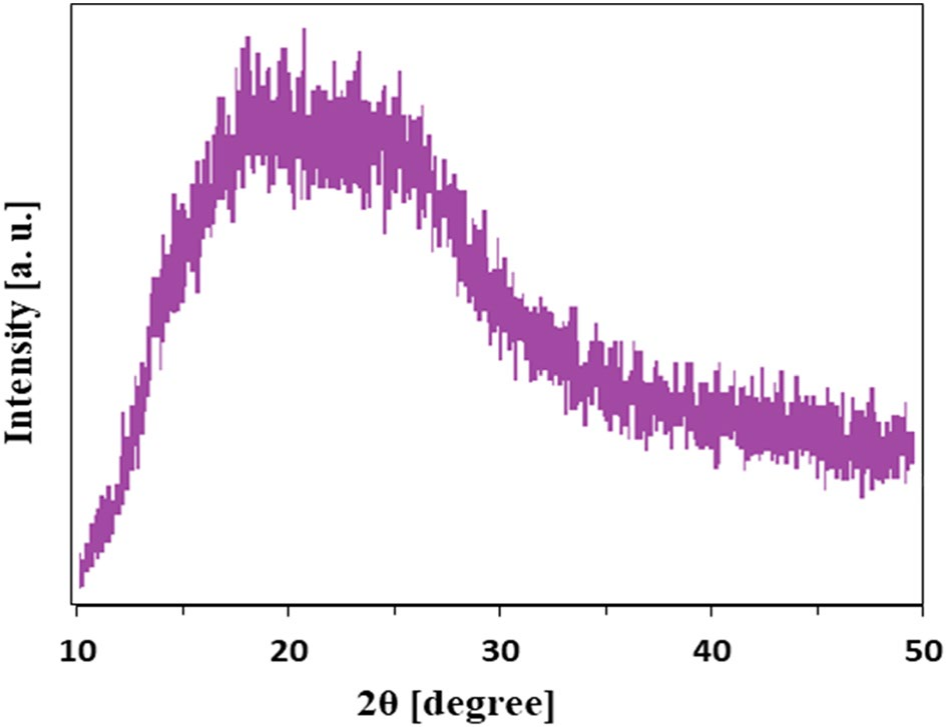
X-ray diffractogram of the PPY-PEI3 hybrid NPs.

The XRD patterns of all the doped PPy and undoped samples showed no sharp peaks. Figure 4**.** illustrates the PPy-PEI3 NPs XRD pattern prepared with an oxidant/pyrrole with a molar ratio of 2 in 1 M HCl at ice bath temperature for 1.5 h and 0.25 g of PEI. The broad peak indicates that the films are amorphous. The PPy NPs pattern exhibits a broad peak from 2θ = 18–26°, with peaks centered at 2θ = 21.5° and 26.0° assigned to the scattering from the PY counterion or inter-counterion interactions and the PY-PY inter-planar distance, indicating successful PY polymerization to PPy^37,38^.

### Morphology study

**Figure 5:**
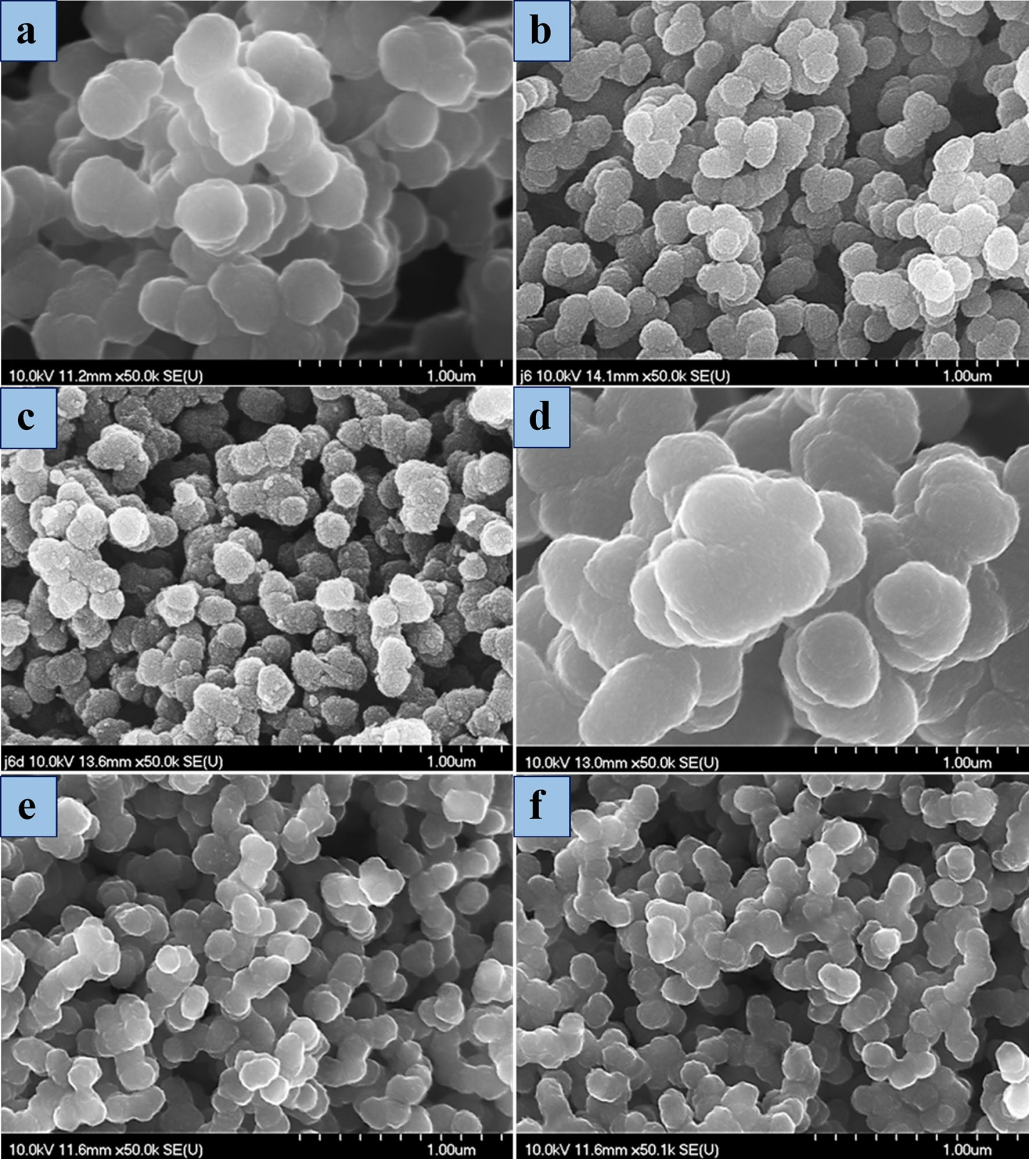
SEM images of as-prepared PPy hybrid NPs with a constant amount of PEI at a temperature of 4 °C for 1.5 h: (**a**) PPy-PEI1, (**b**) PPy-PEI3, (**c**) PPy-PEI3U, (**d**) PPy (Ref), (**e**) PPy-PEI4, (**f**) PPy-PEI5.

**Figure 6:**
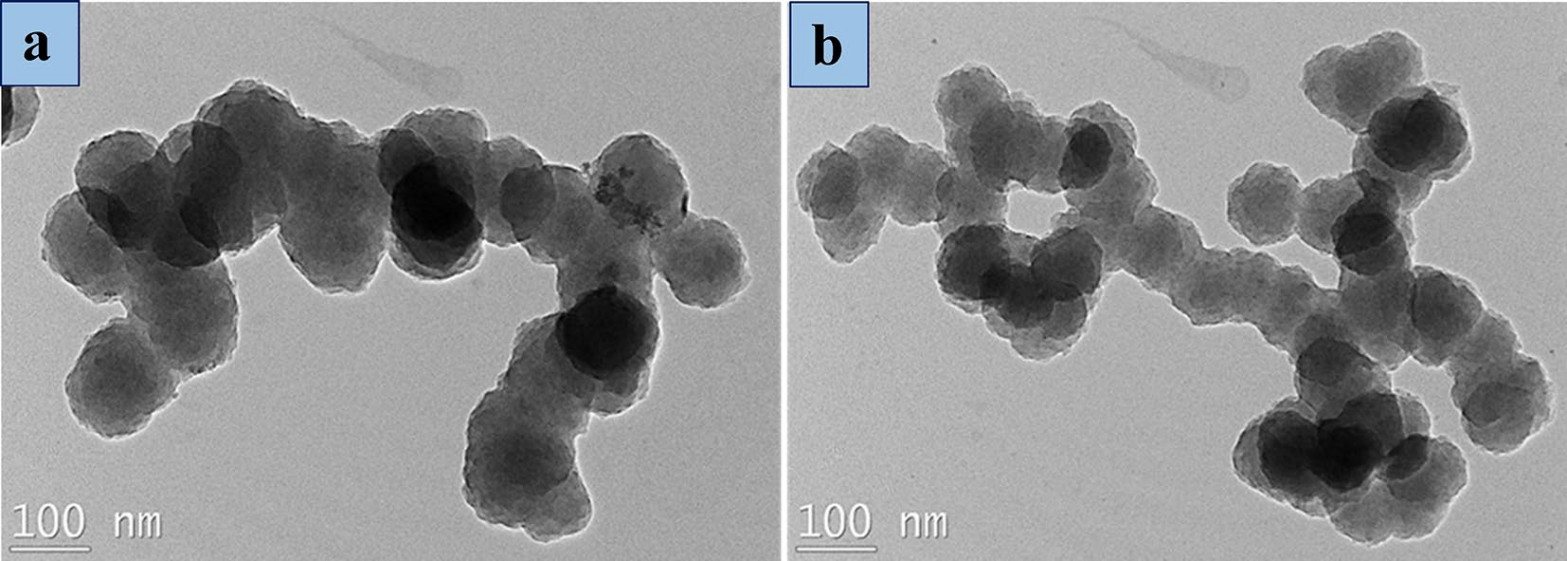
TEM images of as-obtained PPy hybrid NPs; (**a**) PPy-PEI3, (**b**) PPy-PEI5.

The influence of different parameters of the PY polymerization in acidic PEI solutions on the size of the forming PPy hybrid NPs was observed and illustrated in Table 1, Figs. 5, and 6. The capability of PEI to stabilize the aqueous PPy dispersion mainly depends on the PY, oxidant, PEI concentrations, and preparation technique. As can be seen, the final NPs were not stable in water under stirring conditions, but if the polymerization was performed under non-stirred conditions, the PPy NPs were more stable. This phenomenon is related to the agglomeration between the NPs with high interactions (Fig. 5a,b). Smaller, uniform, and spherical NPs were acquired with decreasing PY and oxidant concentration with constant PEI concentration (Figs. 5 and 6, PPy-PEI3 and PPy-PEI5). In stark contrast, if the concentration of reactants (PY, oxidant, and PEI) was reduced, the final NPs also have a smaller size (Fig. 5b,e). Undoping by a NaOH solution led to rougher nanoparticles but an unnoticeable change in particle size (Fig. 5b,c). This change in surface morphology can be attributed to a partial release of the PEI from the particle surface. The best result was obtained for PPy-PEI5, which has more symmetric and smaller particles (85–100 nm). Furthermore, preparing the reference PPy without PEI led to the formation of large NPs and strong agglomeration.

### Electrical conductivity measurement

The conductivity of PPy is affected by both temperature and the concentration of the dopant anion. The electrical conductivity increased to a saturation level with increasing dopant anion concentration. Furthermore, the type of dopant anion used affects the final properties and structure of PPy^39^.

The electrical conductivity of the PPy hybrid NPs and reference PPy NPs (prepared in the absence of PEI) are compiled in Table 1. It should be noted that the conductivity also increased as the oxidant/pyrrole molar ratio increased to 2. This is due to a higher doping level and smaller particle size. The reference PPy NPs had lower conductivity than those prepared under the same conditions, likely due to a higher and more varied particle size distribution. Undoping the hybrid NPs using NaOH solution resulted in the lowest conductivity, while decreasing reactant concentration (PPY-PEI4) and increasing PEI content at the same oxidant/pyrrole molar ratio (PPy-PEI5) led to increased conductivity through smaller and more uniform particle size. Table 1 summarizes the initial conditions, different molar ratios used for hybrid polymer NP preparation, reference polymers, particle size, and electrical conductivity levels.

### UV–vis examination

**Figure 7:**
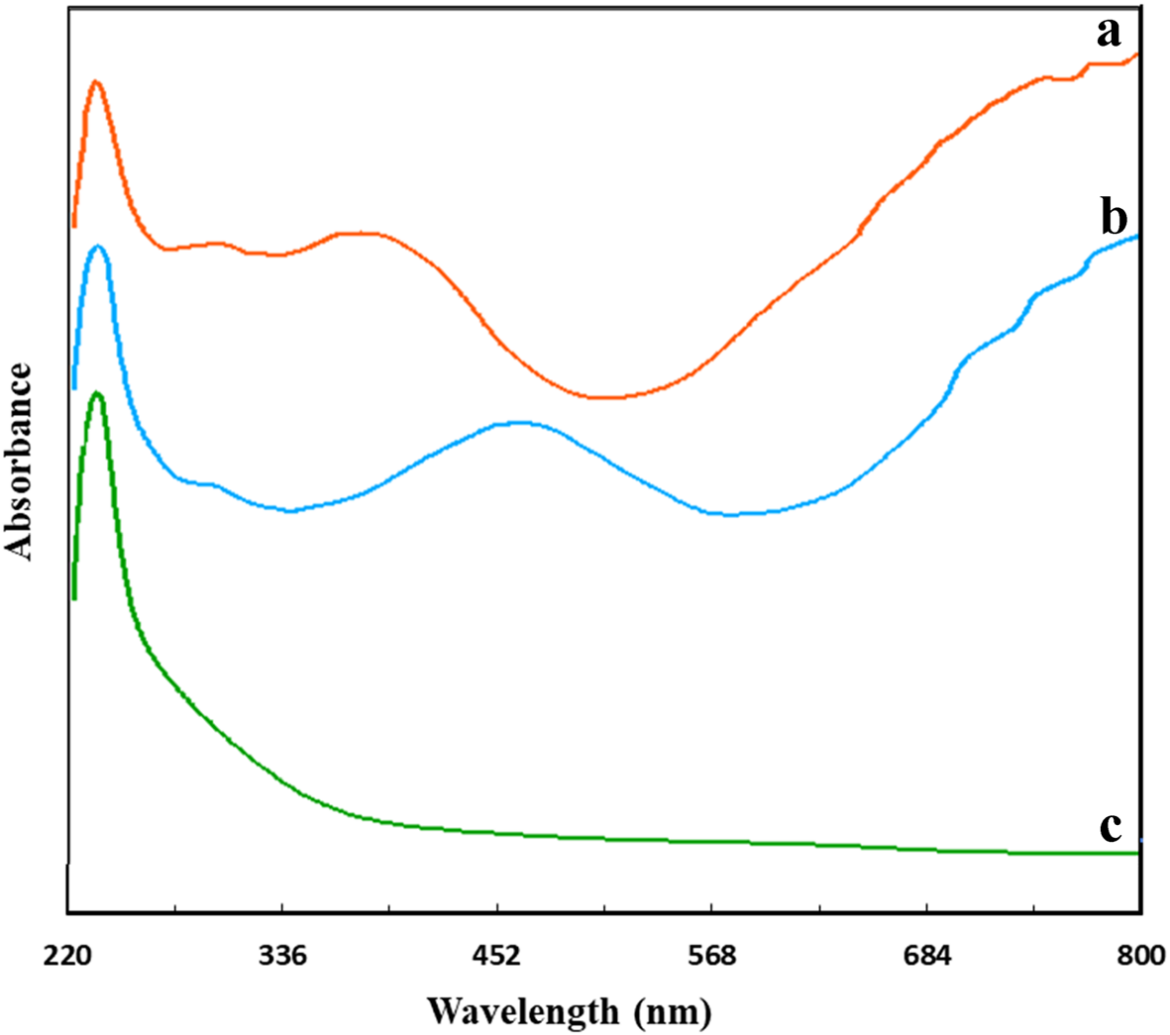
UV–vis spectra of dispersed hybrid PPy NPs (PPy-PEI3) in (**a**) basic, (**b**) acidic, (**c**) PEI in water.

Figure 7 illustrates the UV–vis spectrum of as-prepared specimens in different conditions. The black acidic solution of the NPs (Fig. 7b) exhibits one band at 238 nm corresponding to the amine or imine groups, a broad band centered approximately at 450 nm, and a strong absorption above 800. An intense absorption peak at 420 nm is attributed to the π–π* transition, while a broad absorption band at 850 nm in the near-infrared (NIR) region is associated with polarons and bipolarons, respectively. The 420 nm peak indicates an electron transition from the valence band to the antibonding polaron state, while the absorption beyond 800 nm is a result of electron transition from the valence band to the bipolaron band^40,41^. The bands observed are consistent with those seen for PPy prepared by chemical polymerization.

When the acidic solution of the hybrid polymer is exposed to the base, the color changes to greenish, and the position of the absorption bands shifts. The band at 450 nm shifted to a lower wavelength at about 360 nm, and the other band shifted to near 800 nm. No change in the absorption band of the amine in both acidic and basic solutions was observed. The shift in the peak position is attributed to changes in the doping level on the polypyrrole chains^42^.

### Dependence on pH

**Figure 8:**
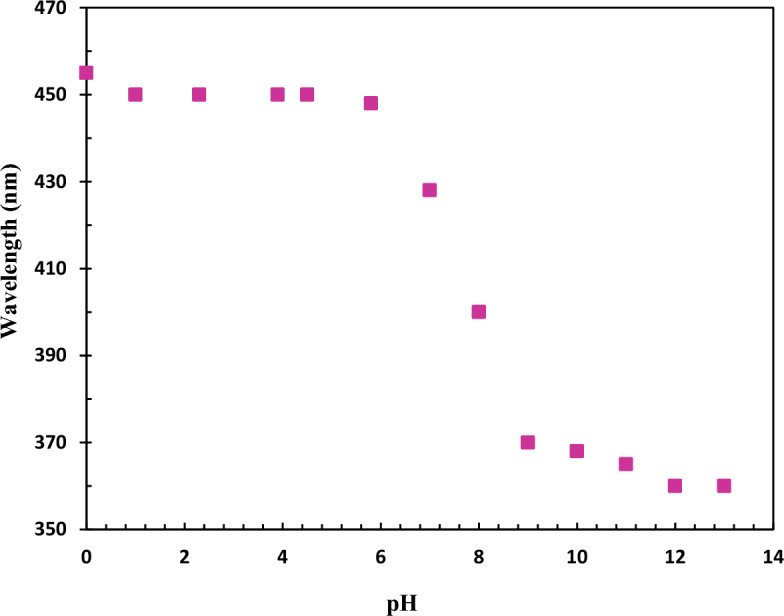
Dependence of the maximum absorption band of PPY-PEI hybrid nanoparticles on the pH of the solution.

All water-dispersed PPy hybrid NPs indicated different colors depending on the acidity or basicity of the solution. Figure 8. shows the change of the maximum absorption band between 350 and 470 nm versus the pH change. The sharp difference in the peak positions at approximately pH = 7 can be attributed to the change in the doping level on the polypyrrole chains. This behavior could be advantageous for the PPy hybrid NPs as an optical pH sensor, restricted for use at pH = 6–10, where the most significant UV–vis spectra changes occur.

### TGA studies

**Figure 9:**
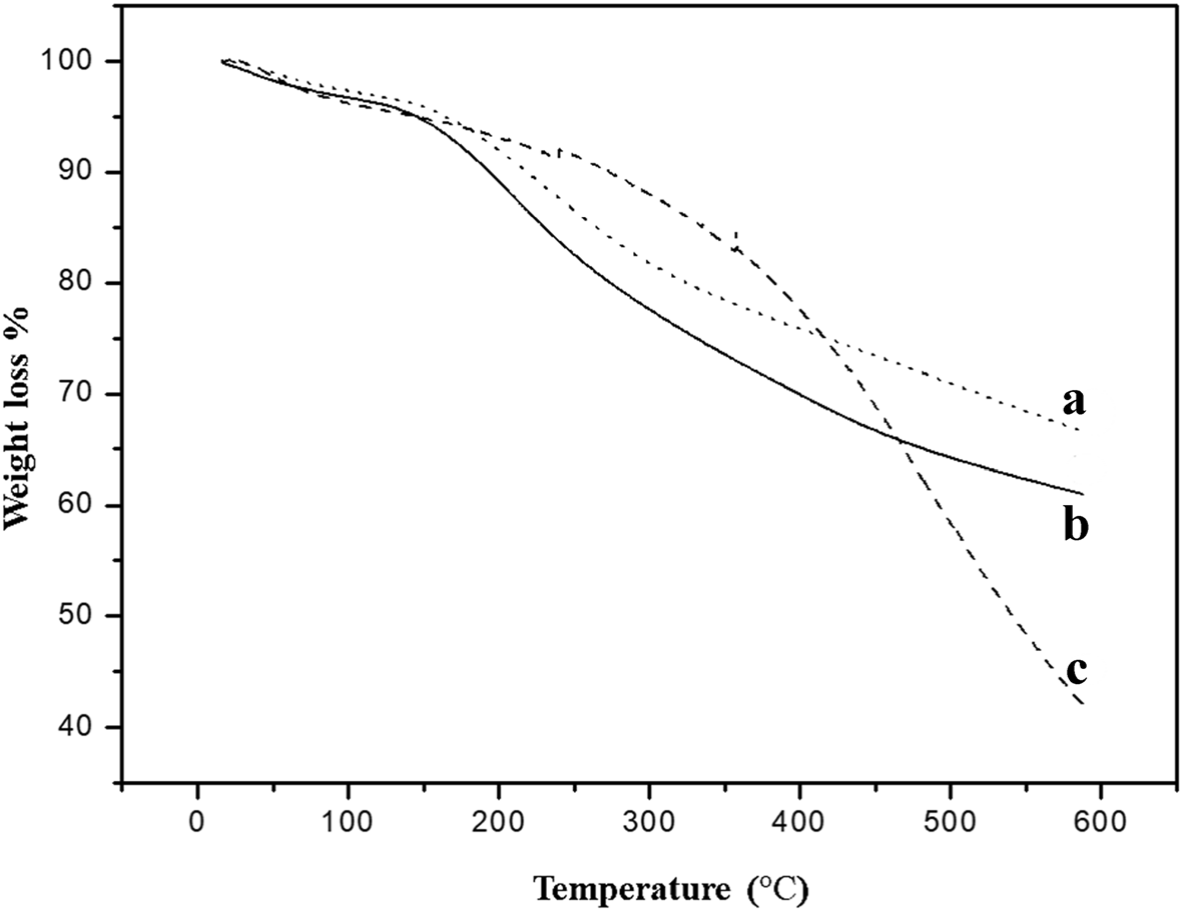
TGA curves of the hybrid nanoparticles (PPY-PEI3) in the doped (**a**) and dedoped (**c**) form and the reference PPy (**b**).

Thermogravimetric analysis (TGA) is a commonly used technique to investigate how materials behave in harsh temperature conditions, including their thermal degradation kinetics^43,44^. In this study, the TGA patterns of reference polypyrrole (PPy) and two hybrid PPy nanoparticles (NPs) in both doped and dedoped forms were examined in a nitrogen atmosphere with a heating rate of 10 °C min^−1^ are exhibited in Fig. 9 to assess the influence of doping and PEI3 on the thermal durability of PPy. All samples were prepared under the same conditions. The reference PPy and PPY-PEI3 samples follow almost similar degradation curves. After removing the moisture from the reference PPy and doped PPy hybrid NPs (Fig. 9a) up to 150 °C, the degradation started at around 200 °C and continued up to 600 °C. The reference PPy (Fig. 9b) prepared in the doped state indicates a gradual degradation trend, while the undoped PPy hybrid NPs (PPy-PEI3) degraded relatively sharply.

On the other hand, in the dedoped PPy hybrid NPs, the amount of residue at around 600 °C decreased as the conducting fraction was lowered (i.e., in the presence of PEI). The dedoped, doped PPy hybrid NPs (PPy-PEI3U) (Fig. 9c) were more stable compared to the reference, and doped forms and the degradation began at a higher temperature (above 250 °C) after the removal of moisture. It continued up to 600 °C with a lower slope in the beginning compared to its dedoped form and fast reduction in mass over 350 °C. However, the final residual mass was less than the others, which could correspond to the facile degradation of the PPy chains after dedoping and decreasing the PEI and anions (dopants) in the final hybrid nanoparticles. It is worth noting that the practical environment (closely resemble real-world) is used as the primary condition for the TGA tests in our study to obtain valuable results under conditions as close as possible to practical applications.

### Discussion about the preparation of PPy nanohybrids

Conditions were established to fabricate nanoparticles that are well dispersed in water. However, when hybrid polymer nanoparticles were produced under stirring conditions, their dispersibility in water was low due to strong particle interaction and subsequent agglomeration. Interestingly, the dispersibility of hybrid PPy NPs decreased with decreasing PEI content and degree of dryness. The reference PPy did not disperse in water even after prolonged ultrasonication. To prepare conductive hybrid materials, the presence of acid in the reaction mixture is essential as polyamine is a strong base with nucleophilic properties. Non-protonated amine groups on the polyamine can form complexes with the oxidant (Fe (III)) or react with propagated polypyrrole oligomers. Attempts to produce the product in deionized water or using protonated PEI and distilled water yielded no positive results. It has been reported that the presence of acid can increase the pyrrole polymerization rate^45^.

### Effect of time and different conditions on particles size

It is possible that the particle size of PPy changes over time, depending on the conditions experienced during synthesis and storage. The concentration of the oxidizing agent, which can influence the rate and degree of polymerization, as well as the formation of cross-links, is one of the factors that can affect particle size over time. To stabilize the dispersion and prevent the particles from aggregating, the type and concentration of the surfactant are both important factors. It is possible that the solubility and stability of the polymer and surfactant are affected by the temperature and pH of the environment in which the reaction and storage processes occur. It is possible for the polymer to undergo degradation or oxidation as a result of exposure to light, oxygen, or moisture, which can also cause changes in its properties. For this reason, it is essential to optimize the synthesis parameters and store the PPy dispersion in cool, dry, and dark conditions. This allowed us to maintain control over the particle size over time.

Second, changing the reaction time affects the particle size of the hybrid PPy nanoparticles. SEM analysis revealed that smaller, uniform, and spherical nanoparticles were obtained when the reactant (pyrrole, oxidant, and PEI) concentrations were reduced. This indicated that decreasing the reaction time could potentially result in smaller particle sizes. Additionally, the analysis showed that the best dispersibility and smaller particles (ranging 85–100 nm) were achieved with a specific PEI concentration (PPy-PEI5). This suggests that the concentration of PEI, which acts as a template and stabilizer, also influences the particle size. Furthermore, PPy prepared in the absence of PEI resulted in large nanoparticles and strong agglomeration, indicating that the presence of PEI plays a significant role in controlling the particle size. In summary, based on the SEM analysis and observations mentioned in the provided information, it can be concluded that changing the reaction time, as well as the concentrations of the reactants and PEI, affects the particle size of the hybrid PPy nanoparticles. Decreasing the reaction time and adjusting the reactant concentration can lead to smaller and more uniform nanoparticles.

### Different dopants and controlling the particles size

The particle size of other dopants can be controlled by adjusting the synthesis parameters and conditions. The choice of dopant can influence the polymerization process and the resulting particle size. Different dopants may have varying effects on the reaction kinetics, stability, and growth of the particles. By carefully selecting and optimizing the dopant concentration, reaction temperature, and reaction time, it is possible to achieve desired particle sizes in different dopant systems. As an illustration, certain dopants have the ability to enhance the crystalline order and electrical conductivity of PPy, as well as improve its solubility and processability in a variety of solvents^46^*.* It is possible for certain dopants, such as FeCl_3_, to function as cross-linking agents and enhance the thermal stability of PPy. To stabilize the dispersion and prevent the particles from aggregating, the type and concentration of the surfactant are both important factors. Some surfactants, such as Triton X-100, SDS, and PEG, for instance, have the ability to decrease the particle size of PPy and improve its colloidal stability^47^. It is the temperature at which polymerization occurs which can have an impact on the rate and degree of polymerization as well as the formation of cross-links. When compared to lower temperatures, for instance, higher temperatures can produce particles that are smaller and more uniform, whereas lower temperatures can produce particles that are larger and more irregular. Therefore, the particle size of PPy with various dopants can be controlled by adjusting the above-mentioned parameters.

### PEI and its role in size controlling

Polyethyleneimine (PEI) plays a crucial role in controlling the size of the hybrid polypyrrole nanoparticles (PPy NPs). PEI acts as a template and amine source during the synthesis process. It interacts with the pyrrole monomers and influences their polymerization and growth behavior. PEI helps to stabilize the aqueous PPy dispersion, preventing agglomeration and promoting the formation of smaller, uniform, and spherical NPs. The presence of PEI on the particle surface enhances the interaction between the particles and the solvent, leading to improved dispersibility. By adjusting the concentration of PEI, along with other parameters such as the concentration of pyrrole monomers and oxidants, it is possible to control the size of the resulting PPy NPs. A decrease in the concentration of PEI can lead to smaller particle sizes, whereas higher concentrations may result in larger particles.

It is possible for the PEI to form a complex with the pyrrole, which functions as a surfactant and contributes to the reduction of the interfacial tension as well as the enhancement of the solubility of pyrrole. This can lead to the formation of a microemulsion-like system that containing a variety of nanostructures, including spherical micelles, worm-like micelles, and vesicle structures^47^. Both the size and the morphology of the PPy nanoparticles are determined by the concentration of PEI and pyrrole, in addition to the temperature and the pH of the solution. Through the provision of charge carriers and the enhancement of charge transport, PEI has the potential to influence the electrical conductivity, thermal stability, and electrochemical performance of PPy nanoparticles^48^. Therefore, PEI has the potential to play a significant role in controlling the particle size and properties of PPy nanoparticles.

## Conclusions

In this study, an unstirred polymerization process for pyrrole was successfully utilized to produce dispersible and conductive hybrid PPy nanoparticles. The hybrid PPy-PEI nanoparticles were prepared through an oxidative polymerization process at low temperatures in an acidic solution, utilizing PEI as a template and amine source. The variations in oxidant, monomer, and PEI concentrations had a significant impact on the size, shape, electrical conductivity, and dispersibility of the hybrid PPy nanoparticles, acting as both steric stabilizers and templates. The consistent yield and diameter of the produced PPy nanoparticles were observed throughout the experiments.

The hybrid PPy nanoparticles exhibited bulk electroconductivities ranging from 0.036 to 6.9 S·cm^−1^, showcasing their potential for various applications. Furthermore, the ability of these hybrid PPy nanoparticles to modulate their absorption in the UV–vis range in response to pH renders them promising candidates for optical sensors. Additionally, the inclusion of amines in the hybrid nanoparticles enhances their potential as efficient bioadsorbents and vectors for gene delivery.

Overall, the conducting polymer nanoparticles developed in this study show promise as effective trapping vectors for the removal of a diverse range of organic and metallic pollutants, highlighting their potential impact in environmental remediation and biomedical applications.

## Data Availability

All data generated or analyzed during this study are included in this published article.
